# Evaluation of the force generated by gradual deflection of orthodontic wires in conventional metallic, esthetic, and self-ligating brackets

**DOI:** 10.1590/1678-775720150405

**Published:** 2016

**Authors:** Manoela Fávaro Francisconi, Guilherme Janson, José Fernando Castanha Henriques, Karina Maria Salvatore de Freitas

**Affiliations:** Universidade de São Paulo, Faculdade de Odontologia de Bauru, Departamento de Odontopediatria, Ortodontia e Saúde Coletiva, Bauru, SP, Brasil.

**Keywords:** Orthodontic wires, Mechanical phenomena, Elasticity, Comparative study

## Abstract

**Objective:**

The purpose of this study was to evaluate the deflection forces of Nitinol orthodontic wires placed in different types of brackets: metallic, reinforced polycarbonate with metallic slots, sapphire, passive and active self-ligating, by assessing strength values variation according to gradual increase in wire diameter and deflection and comparing different combinations in the different deflections.

**Material and Methods:**

Specimens were set in a clinical simulation model and evaluated in a Universal Testing Machine (INSTRON 3342), using the ISO 15841 protocol. Data were subjected to One-way ANOVA, followed by Tukey tests (p<0.05).

**Results:**

Self-ligating brackets presented the most similar behavior to each other. For conventional brackets there was no consistent behavior for any of the deflections studied.

**Conclusions:**

Self-ligating brackets presented the most consistent and predictable results while conventional brackets, as esthetic brackets, showed very different patterns of forces. Self-ligating brackets showed higher strength in all deflections when compared with the others, in 0.020-inch wires.

## INTRODUCTION

Nowadays, having a natural and pleasant smile even during orthodontic treatment is one of patients' main concerns. Devices combining acceptable esthetic and adequate technical performance, satisfying both the patient and the clinician expectations, have been developed^2^. Nevertheless, esthetic brackets show higher friction coefficients than metallic brackets, which can impair the desired movement^35^.

Self-ligating brackets, introduced as Russel's accessories in the mid-1930s, are systems that present a mechanical device designed to close the edgewise slot^33^. Their use has become common in recent years. Manufactures claim several advantages in using these accessories, and the low friction seems to be the most studied among them^11,15,18^. Some studies confirm that there is significantly lower friction in these brackets, increasing the efficiency of the alignment, resulting in a shorter treatment time^15,19^. However, some studies showed that this reduced friction depends on the type and caliper of the wire and the degree of crowding^30^. Further studies and clinical evaluations are still necessary to confirm these benefits^24^.

Therefore, effectiveness of orthodontic movement results not only from different bracket systems, but also from a series of other factors, related to both the patient (teeth and supporting structures) and the type of mechanics applied. Teeth movement also depends on the action of orthodontic wires, which varies according to their structural and mechanical properties^3^.

Consequently, it is necessary to assess not only the friction related to different bracket systems, but also the behavior of different currently available orthodontic materials regarding the forces applied during orthodontic mechanics. Furthermore, the development of esthetic brackets with metal components comprises a new field for research^8^. To make the best choice among the various brackets and orthodontic wires available, it is essential to know the magnitude of forces released by these wires and their behavior regarding the gradual increase in wire deflection^7^.

This *in vitro* study assessed deflection forces of round Nitinol orthodontic wires, placed in conventional metallic, reinforced polycarbonate, sapphire (Ormco, Glenda, CA, USA), and self-ligating brackets by using a clinical simulation model and following ISO 15841 as protocol.

## MATERIAL AND METHODS

### Material - experimental groups

**Figure 1:**
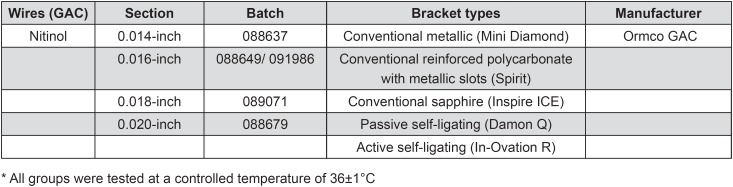
Sample used and test conditions

The sample used in this study consisted of 400 round-section Nitinol wires (Standard or Medium, GAC^®^, Bohemia, NY, USA) with 0.014, 0.016, 0.018, and 0.020-inch diameters and five different bracket types: conventional metallic, reinforced polycarbonate with metallic slots, sapphire, passive and active self-ligating brackets (Figure 1). Wires that showed very different behavior from the others were excluded^21^: they statistically represent outliers that would negatively interfere with the results. In conventional brackets, the wires were tied with “O” shaped elastomeric ligatures (GAC^®^; Bohemia, NY, USA). In the self-ligating brackets the wires were tied by closing the passive (Damon Q) or active (In-Ovation R) systems.

### Methods - clinical simulation device

In order to internationally standardize the tests as adequately as possible, the methods used in this study followed the ISO 15841 Standard: Dentistry – Wires for use in orthodontics^16^.

**Figure 2:**
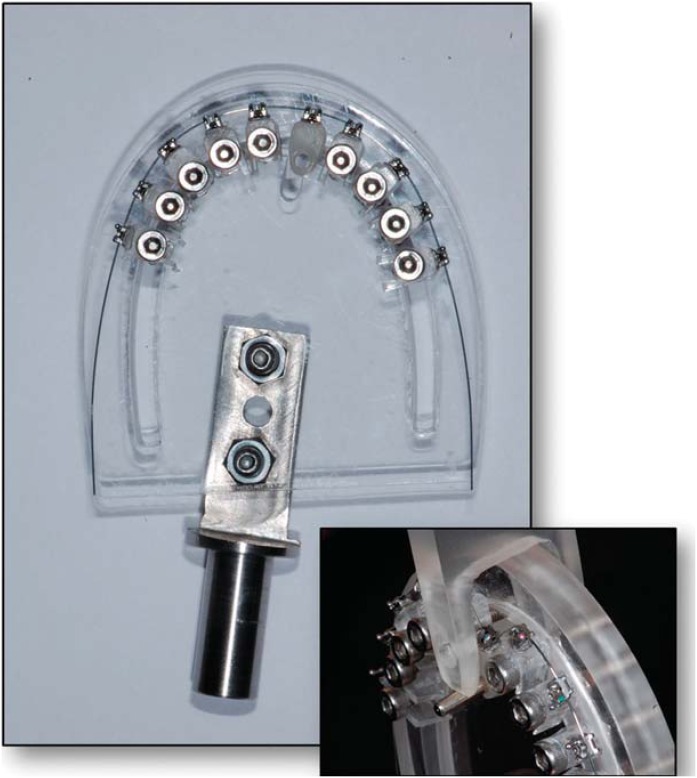
Clinical simulation device

Deflection of the orthodontic wire was performed in a clinical simulation device representing all 10 teeth of the maxillary arch^11^. Figure 2 shows the clinical simulation device that was used in this study. This device consists of a parabola shaped acrylic resin plate with fixed acrylic structures representing the maxillary teeth (Figure 2). The parabola shape was determined by the wires, reducing the risk of diverse forces arising from the deflection applied in an unexpected way. Brackets were bonded with cyanoacrylate ester gel (Super Bonder, Loctite, São Paulo, SP, Brazil), positioned in the long axis of the acrylic devices parallel to the ground and at the same height. The wires had the same length^12^. The acrylic structures were fixed by means of threaded screw in the bottom of the acrylic resin plate.

**Figure 3:**
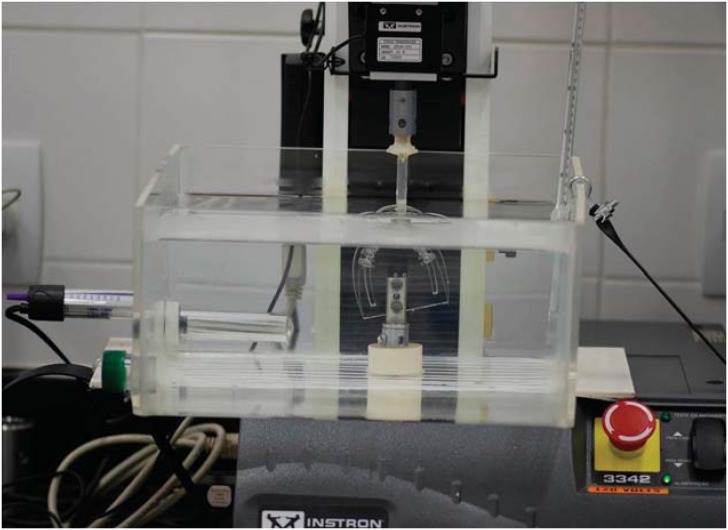
Instron universal testing machine used in this study, with a load cell of 10 N. Acrylic container adapted to the Instron device

The tests were performed on the structure corresponding to the right maxillary central incisor (Figure 3). Unlike the others, this structure was not screwed, enabling its labio-lingual movement. It had a perforation, in which a metal cylinder was placed to activate it. The tip of the activation head, attached to the testing machine, had a rounded cut to fit the metal cylinder. Deflection of the wire was performed without changing the inter bracket distance (6 mm), since the relation deflection/force depends, among other things, on this distance. The speed of the deflection was 2 mm/min.

Records of the force released by the wire deflection were made in 0.5, 1, 2, and 3 mm. Deflection of the wire attached to the bracket clinically corresponds to the beginning of treatment, when the teeth are poorly positioned and the wire is forced into the accessories slots. Depending on the degree of crowding, teeth experience more or less force to align the teeth.

The deflection tests were performed using the Universal Testing Machine (Instron 3342), with a load cell of 10 N^10^ (Figure 3). This load cell has an accuracy of 0.5% of the reading value with the temperature of 25°C. In this study, the load cell was maintained at this temperature. Also according to the ISO standard, the tests were always performed at the same testing temperature of 36±1°C for all test groups^26^. To obtain this, an acrylic container with water at a temperature of 36±1°C, maintained with the aid of submersible heater with integral thermostat (Electronic Atman Heater, China) and checked by a decimal precision thermometer, with a limit of error of ± 0.2°C (Incoterm, reference 5097, São Paulo, SP, Brazil), was adapted to the testing machine^24^ (Figure 3).

### Statistical analyses

The sample size, according to the ISO 15841 standards, is of six specimens in each group^16^. To minimize the chances of any technical error and increase reliability of the results, 20 specimens were chosen for each group. Outliers were excluded through a statistical program that provides the values to be deleted^22^. Normal distribution was evaluated with Kolmogorov-Smirnov tests. Because all variables showed a normal distribution, one-way ANOVA and Tukey tests were used.

All statistical analyses were performed with Statistica software (Statistica for Windows – Release 7.0 - Copyright Statsoft Inc., Tulsa, OK, USA). Results were considered significant at p<0.05.

## RESULTS

The deactivation forces were generally significantly higher with conventional than with self-ligating brackets, with 0.014 and 0.016-inch nickel-titanium wires, except with the activation of 2 mm, in which the opposite occurred (Tables 1 and 2). Overall, Inspire ICE showed the highest deactivation forces while In-Ovation R showed the lowest.

**Table 1:** Deactivation forces (cN) comparisons of the bracket types with 0.014-inch nickel-titanium wire, in progressive deflections (One-way Anova followed by Tukey tests)

Deflection (mm)	Mini Diamond (N=15)	Spirit (N=20)	Inspire ICE (N=17)	Damon Q (N=16)	In-Ovation R (N=16)	P
	Mean (SD)	Mean (SD)	Mean (SD)	Mean (SD)	Mean (SD)	
0.5	123.78 (22.70)^A^	60.13 (32.77)^B^	97.23 (41.87)^A^	61.08 (5.61)^B^	39.15 (9.46)^B^	<0.001
1.0	143.27 (10.56)^AD^	118.32 (8.74)^B^	149.73 (16.70)^A^	135.10 (5.96)^D^	100.27 (10.21)^C^	<0.001
2.0	76.57 (10.36)^A^	92.63 (15.15)^B^	91.74 (20.94)^B^	156.59 (9.45)^C^	153.56 (10.02)^C^	<0.001
3.0	391.28 (18.28)^A^	341.65 (15.03)^B^	498.63 (32.73)^C^	204.24 (9.52)^D^	190.19 (9.38)^D^	<0.001

Different letters represent statistically significant differences

**Table 2:** Deactivation forces (cN) comparisons of the bracket types with 0.016-inch nickel-titanium wire, in progressive deflections (One-way Anova followed by Tukey tests)

Deflection (mm)	Mini Diamond (N=19)	Spirit (N=17)	Inspire ICE (N=17)	Damon Q (N=18)	In-Ovation R (N=20)	P
	Mean (SD)	Mean (SD)	Mean (SD)	Mean (SD)	Mean (SD)	
0.5	112.97 (65.22)^A^	59.69 (43.36)^B^	130.14 (53.65)^A^	86.58 (4.80)^B^	70.72 (16.22)^B^	<0.001
1.0	231.49 (26.93)^AD^	184.01 (20.76)^B^	248.30 (24.89)^A^	226.47 (12.22)^D^	162.65 (19.95)^C^	<0.001
2.0	218.93 (32.20)^A^	224.54 (25.33)^A^	232.22 (21.29)^A^	281.85 (15.04)^B^	273.24 (15.80)^B^	<0.001
3.0	479.55 (31.76)^A^	459.29 (18.29)^A^	569.15 (38.27)^B^	372.87 (12.22)^C^	353.17 (21.67)^C^	<0.001

Different letters represent statistically significant differences

The deactivation forces were generally significantly higher with conventional than with self-ligating brackets, with 0.018-inch nickel-titanium wires (Table 3). Overall, Inspire ICE showed the highest deactivation forces while In-Ovation R showed the lowest.

**Table 3:** Deactivation forces (cN) comparisons of the bracket types with 0.018-inch nickel-titanium wire, in progressive deflections (One-way Anova followed by Tukey tests)

Deflection (mm)	Mini Diamond (N=16)	Spirit (N=19)	Inspire ICE (N=14)	Damon Q (N=18)	In-Ovation R (N=19)	P
	Mean (SD)	Mean (SD)	Mean (SD)	Mean (SD)	Mean (SD)	
0.5	158.85 (21.14)^A^	101.08 (53.22)^B^	141.52 (43.53)^AC^	108.70 (9.86)^BC^	96.85 (16.57)^B^	<0.001
1.0	289.29 (17.81)^AD^	236.63 (26.26)^B^	269.06 (17.09)^A^	304.09 (12.66)^D^	209.14 (21.53)^C^	<0.001
2.0	400.40 (31.08)^A^	351.94 (18.07)^B^	397.88 (37.32)^A^	392.24 (15.60)^C^	354.15 (22.05)^B^	<0.001
3.0	626.94 (27.36)^A^	588.17 (32.19)^B^	674.28 (31.55)^C^	538.04 (17.69)^E^	486.04 (38.02)^D^	<0.001

Different letters represent statistically significant differences

There was an inversion of the previous tendency with 0.020-inch nickel-titanium wires. The deactivation forces were generally significantly higher with self-ligating than with conventional brackets (Table 4). Overall, Damon Q showed the highest deactivation forces while Spirit showed the lowest. The forces with the Damon Q with 3 mm of activation exceeded 1000g.

**Table 4:** Deactivation forces (cN) comparisons of the bracket types with 0.020-inch nickel-titanium wire, in progressive deflections (One-way Anova followed by Tukey tests)

Deflection (mm)	Mini Diamond (N=16)	Spirit (N=18)	Inspire ICE (N=19)	Damon Q (N=18)	In-Ovation R (N=20)	P
	Mean (SD)	Mean (SD)	Mean (SD)	Mean (SD)	Mean (SD)	
0.5	130.12 (34.33)^A^	58.56 (46.10)^B^	127.83 (66.48)^A^	159.03 (17.74)^A^	145.61 (26.12)^A^	<0.001
1.0	249.64 (34.96)^A^	210.17 (26.03)^B^	259.66 (30.11)^A^	423.59 (36.33)^D^	344.09 (25.04)^C^	<0.001
2.0	560.60 (20.07)^A^	410.95 (41.75)^B^	550.04 (39.91)^A^	774.27 (77.22)^D^	620.34 (52.04)^C^	<0.001
3.0	804.86 (29.18)^A^	753.09 (24.42)^B^	815.67 (21.90)^A^	**–––––**	858.98 (84.34)^A^ **	<0.001

Different letters represent statistically significant differences

** n=5 because the other wires exceeded the force of 1000g

## DISCUSSION

### Sample and methodology

A clinical simulation device was used to approximate the laboratory results to clinical situations, providing more practical applications^32^. Even with this in mind, the specific ISO standard was used for orthodontic wires laboratory tests^16^.

The elastic deflection test was chosen because it is clinically closest to the orthodontists' interests, since that is what they do when adapting a wire to the patient's teeth. Although engineers work with parameters such as elastic modulus and yield value, the orthodontist is more concerned about knowing the force released regarding the amount of deflection.

### Results

In general, it was observed that the deactivation force increased with the increase in amount of deflection (Tables 1 to 4). These findings are consistent with other studies^29^.

The results found in different combinations of the brackets with 0.014-inch nickel-titanium wires are in agreement with other studies in the literature that have found erratic results in different deflections, large variation in the difference of force values, and significant differences between different types of devices^36^ (Table 1).

The self-ligating brackets, in deflection of 2 mm, presented the highest forces when compared with conventional brackets (Table 1). This result can be explained by assuming that part of the force is used to overcome the greater resistance to sliding, generated in tests with conventional bracket systems during unloading^6,34^. These findings are grounded in previous studies and confirm that NiTi wires, along with self-ligating bracket systems, generate significantly higher forces when compared with conventional brackets^1,9^.

However, in deflection of 3 mm, the self-ligating brackets presented the smallest forces, contradicting previous investigation^27^ (Table 1). This occurs because smaller diameter wires release smaller forces, since they are not completely pressed against the slots. In conventional brackets, even small diameter wires are pressed into the slots by the elastic tie.

Confirming previous results^31^, there were no differences in the discharge forces between Damon and In-Ovation self-ligating brackets with 0.014-inch NiTi wires in most deflections (Table 1). This may be a consequence of the more uniform wire mechanical locking system than the wire tying process of conventional brackets, with elastomeric ligatures^36^. There may be more variation in the process of tying the elastomeric ligatures than when closing the self-ligating brackets.

The results were not standardized between different bracket combinations with 0.016-inch Nitinol wires, showing that the design of the experimental testing qualitatively and quantitatively affects the discharge forces during leveling and alignment with superelastic NiTi wires^28,36^ (Table 2). Similarly, when these forces were evaluated in an experimental model with embedded bracket systems, the wires tended to lose their superelastic properties, showing variations of force^27^. Other studies confirmed these findings^24,25^.

The self-ligating brackets continued delivering less force in the 3 mm deflection because of the reasons previously provided (Table 2).

The results of combinations of different bracket types with 0.018-inch Nitinol wires demonstrated that Damon Q generated significantly greater forces when compared with In-Ovation R (Table 3). Other studies^4,21^ also noticed higher forces when passive (Smart Clip) were compared with active self-ligating brackets (Time3).

Finally, different bracket combinations with 0.020-inch Nitinol wires showed again that Damon Q generated greater forces than In-Ovation R (Table 4). From 1 mm of deflection, the self-ligating brackets showed greater forces than the others. This result is consistent with another study in which friction was responsible for reducing the amount of released force^36^. This partially explains the fact that the highest average was generated by the simulation device with self-ligating brackets^14,17^.

Clinically, this explanation makes sense because friction increases the released force during loading, but decreases it during unloading^23^. Therefore, the device with higher friction generated less force, because friction hinders the return of the wire to its initial position during the discharge^36^. The presence of the brackets, the distance between them, the bands, and the crowding itself are factors that increase friction in the clinical setting. Thus, this large friction would be able to decrease the released force by the wire. Other studies also found higher frictional resistance of conventional brackets when compared with self-ligating ones^5,13^.

Conventional and esthetic brackets showed very different patterns of forces due to bracket composition. Thus, this result should be considered when choosing the type of brackets to be used according to the type of mechanics necessary in orthodontic treatment of each case.

An important aspect to be considered is the difficulty in extrapolating laboratorial findings regarding frictional forces to the clinical environment. Many different factors, such as occlusal forces and tightening or loosening the archwire in the brackets during treatment, may produce different force values, with significant clinical relevance^20^.

## CONCLUSIONS

Conventional and esthetic brackets showed very different patterns of forces due to bracket composition;

Self-ligating brackets presented similar behavior to each other and showed higher strength in all deflections when compared with the others, in 0.020-inch wires.
